# Phenotypic flexibility in heat production and heat loss in response to thermal and hydric acclimation in the zebra finch, a small arid-zone passerine

**DOI:** 10.1007/s00360-020-01322-0

**Published:** 2020-10-18

**Authors:** Michał S. Wojciechowski, Anna Kowalczewska, Roger Colominas-Ciuró, Małgorzata Jefimow

**Affiliations:** 1 Department of Vertebrate Zoology and Ecology, Faculty of Biological and Veterinary Sciences, Nicolaus Copernicus University, Toruń, Poland; 2 Department of Animal Physiology and Neurobiology, Faculty of Biological and Veterinary Sciences, Nicolaus Copernicus University, Toruń, Poland

**Keywords:** Energy metabolism, Evaporative heat loss, Thermoregulation, Phenotypic flexibility, Passerine

## Abstract

To maintain constant body temperature (*T*_b_) over a wide range of ambient temperatures (*T*_a_) endothermic animals require large amounts of energy and water. In hot environments, the main threat to endothermic homeotherms is insufficient water to supply that necessary for thermoregulation. We investigated flexible adjustment of traits related to thermoregulation and water conservation during acclimation to hot conditions or restricted water availability, or both, in the zebra finch, *Taeniopygia guttata* a small arid-zone passerine. Using indirect calorimetry, we measured changes in whole animal metabolic rate (MR), evaporative heat loss (EHL) and *T*_b_ before and after acclimation to 23 or 40 °C, with different availability of water. Additionally, we quantified changes in partitioning of EHL into respiratory and cutaneous avenues in birds exposed to 25 and 40 °C. In response to heat and water restriction zebra finches decreased MR, which together with unchanged EHL resulted in increased efficiency of evaporative heat loss. This facilitated more precise *T*_b_ regulation in heat-acclimated birds. Acclimation temperature and water availability had no effect on the partitioning of EHL into cutaneous or respiratory avenues. At 25 °C, cutaneous EHL accounted for ~ 60% of total EHL, while at 40 °C, its contribution decreased to ~ 20%. Consistent among-individual differences in MR and EHL suggest that these traits, provided that they are heritable, may be a subject to natural selection. We conclude that phenotypic flexibility in metabolic heat production associated with acclimation to hot, water-scarce conditions is crucial in response to changing environmental conditions, especially in the face of current and predicted climate change.

**Electronic supplementary material:**

The online version of this article (10.1007/s00360-020-01322-0) contains supplementary material, which is available to authorized users.

## Introduction

Endothermic homeothermy is the ability to maintain relatively constant body temperature over the wide range of ambient temperatures (*T*_a_) using behavioral and physiological mechanisms of heat production and heat dissipation. In the cold, maximum heat production in resting animals may be as high as nine times basal heat production (Swanson 2010), while in the heat, when operative (Bakken 1992) or air temperature exceeds body temperature (*T*_b_), birds can dissipate as much as five times as much heat than is produced (O'Connor et al. 2017). However, under such conditions, heat dissipation is possible only via evaporation of water from the body surfaces, both respiratory and cutaneous (for example, Marder and Arieli 1988; McKechnie and Wolf 2004b; Williams and Tieleman 2005). Effective evaporation enables birds to maintain thermal balance even during prolonged acclimation to temperatures as high as 60 °C (Marder and Arieli 1988). Water necessary for thermoregulation is obtained both by intake (drinking and food) and as a byproduct of metabolic processes (metabolic water production) (Jenni-Eiermann and Jenni 2012; Schmidt-Nielsen 1997). Passerines can rely on metabolic water production both during flight in desiccating conditions (Gerson and Guglielmo 2011) as well as during rest when fasting (Rutkowska et al. 2016).

In face of increased probability of unpredictable extreme weather events and increased mean ambient temperatures (IPCC 2014; Meehl and Tebaldi 2004), it was predicted that one of the main threats to endothermic homeotherms is insufficient water supply necessary for thermoregulation as well as the direct risk of lethal hyperthermia (Conradie et al. 2020; McKechnie and Wolf 2010). This indeed seems to be the case for mammals and birds living in subtropical areas (Conradie et al. 2019, 2020; McKechnie and Wolf 2010; Welbergen et al. 2008). However, in response to rapid, often unpredictable changes in abiotic conditions, animals flexibly adjust their physiology to match changes in energy demands (Piersma and Van Gils 2011). Thus, it is legitimate to ask whether acclimation or acclimatization to desiccating conditions may mitigate the risk of dehydration and overheating in response to extreme weather events. In several lark species acclimation to *T*_a_’s within their thermoneutral zone (TNZ) led to decreased basal metabolic rate (BMR) and total evaporative water loss (TEWL), and these changes were species specific (Tieleman et al. 2003; Williams and Tieleman 2000). Larks inhabiting desert areas had lower BMR, TEWL and a lower heat transfer coefficient than birds inhabiting mesic areas (Tieleman et al. 2002a). The very few studies of physiological adjustments of thermoregulation in small passerines indicate that seasonally increased ambient temperatures, often exceeding body temperature, also increased efficiency of evaporative heat loss (EHL) which facilitated maintenance of constant *T*_b_ at high *T*_a_’s (Noakes et al. 2016; Oswald et al. 2018). The most striking example of the effect of acclimation to high *T*_a_ on the ability to maintain heat balance in birds comes from the classic studies by Marder and co-workers who showed that rock pigeons (*Columba livia*) flexibly adjusted heat loss by means of evaporation to regulate their *T*_b_, which allows them to live and successfully reproduce under extreme desert heat, provided water is available ad libitum (Dawson 2003; Marder 1983; Marder and Arieli 1988; Marder and Gavrieli-Levin 1987). By increasing cutaneous evaporative water loss (CEWL) rock pigeons protect themselves from hyperthermia, without any noticeable increase in metabolic heat production (Marder and Arieli 1988). It should be noted that Marder and co-workers acclimated rock pigeons to *T*_a_’s exceeding their *T*_b_. Similar flexible adjustments of CEWL were also reported in other Columbiformes (McKechnie and Wolf 2004b). However, if drinking water is limited, increased EWL in the heat can lead to greater risk of dehydration. Therefore, it is not surprising that arid-zone larks at moderate *T*_a_’s had both reduced CEWL and respiratory evaporative water loss (REWL) than mesic species (Tieleman and Williams 2002), yet at high *T*_a_’s, this difference disappeared.

Acclimation of house sparrows *Passer domesticus* to desiccating conditions led to reduced CEWL accompanied by changes in lipid composition in the epidermal *stratum corneum* (Muñoz-Garcia et al. 2008). These results concur with the considerable reduction in total EWL observed in water-deprived zebra finches (*Taeniopygia guttata*), a small arid-zone passerine native to Australia (Zann 1996), exposed to moderately high *T*_a_’s (Calder 1964), as well as with a reduction in CEWL in rock pigeons deprived of water for 48 h (Arad et al. 1987). More detailed knowledge of flexible adjustments in physiology of heat production and dissipation is of key importance for our understanding of the threats of global environmental changes on animal fitness.

In the light of above, we asked what is the pattern of changes in physiology of thermoregulation in small passerine birds in response to prolonged acclimation (> 30 days) to hot or desiccating conditions, or both. To address this question, we used zebra finches as a model species. We predicted that in response to high *T*_a_ (40 °C during the active phase, α-phase) their metabolic rate (MR) decreases compared to birds acclimated to a *T*_a_ below thermoneutrality (23 °C; reported TNZ of the species is between 30 and 40 °C; Calder 1964, but see present results). Moreover, we expected that desiccating conditions (water deprived for half of the α-phase) under both thermal regimes would result in a greater reduction in TEWL than in birds with unlimited access to water. We assumed that the efficiency of heat dissipation in zebra finches with free access to drinking water at high *T*_a_’s would be higher than in those deprived of water and that the threshold for hyperthermia is higher in birds with water available ad libitum. To test these predictions, we measured gas exchange, EWL and *T*_b_ before and after acclimation to hot and desiccating conditions. Finally, we aimed to determine whether prolonged acclimation to the above-described conditions would result in changes in the partitioning of TEWL between the respiratory and cutaneous avenues and predicted that in birds acclimated to restricted water availability under both thermal regimes CEWL is lower than in individuals with ad libitum access to water. To test this prediction, we measured CEWL and REWL, at *T*_a_'s below and above thermoneutrality.

## Materials and methods

### Animals and experimental design

We used 36 adult (2 years old), male zebra finches *Taeniopygia guttata*, Vieillot 1817 originating from the breeding colony at the Max-Planck Institute for Ornithology Seewiesen, Germany. Birds were transferred to the animal facilities at the Nicolaus Copernicus University in Toruń (Poland) ~ 1.5 months prior to the experiments. Throughout the experiment, birds were kept indoors under 12 h photoperiod (lights on at 08:00) in four flight cages (1.22 m × 1.22 m × 1.82 m) in groups not exceeding ten individuals per cage. Prior to and throughout the experiment, birds were fed commercial mix for small exotic graminivores (Mała egzotyka, Karma Mix, Bieruń Nowy, Poland) ad libitum*,* supplemented every other day with fresh greens, hard-boiled egg, eggshells and a vitamin and amino acid mixture (Biosupervit, Biofactor, Skierniewice, Poland) added to the drinking water. At least 20 days before respirometry measurements, a miniature thermosensitive PIT tag (BioTherm 13, Biomark, Boise, ID, USA) was implanted intraperitoneally in each bird, which was later used to measure *T*_b_ with a remote reader (HPR plus, Biomark, Boise, ID, USA). Prior to implantation, PIT tag readings were calibrated against a precision mercury-in-glass thermometer in a controlled temperature water bath at *T*_a_’s between 30 and 48 °C. All procedures were approved by the Local Committee for Ethics in Animal Research in Bydgoszcz (permit 9/2018 and 26/2018).

During initial acclimation, all birds were kept at constant *T*_a_ of 23 ± 2 °C with unrestricted access to water. After the first series of respirometry measurements, birds were assigned randomly to four experimental groups. Eighteen birds were acclimated for ~ 1 month to a constant *T*_a_ of 23 °C, day and night (henceforth 23 °C). Of these, eight individuals had access to water ad libitum and ten were water deprived for 6 h, from 11:00 till 17:00 each day. For the 3 h in the morning and 3 h in the afternoon, access to water was unlimited. The remaining 18 finches were acclimated to 40 °C during the α-phase and 23 °C during *ρ*-phase (inactive) (henceforth 40 °C). At these *T*_a_’s, a group of nine birds was water deprived for 6 h (as the group at 23 °C) and the other nine individuals had access to water *ad lib*. During the course of acclimation, each week the birds were weighed to ± 0.1 g with an electronic balance (SPU402; Ohaus, USA) to monitor changes in body mass (*m*_b_). Body mass was also measured before and after each respirometry trial.

### Whole-body respirometry

After initial acclimation and then after acclimation to a different *T*_a_ and water regime (henceforth: experimental acclimation), the birds' metabolic rates were measured by indirect calorimetry using an open flow respirometry system (Sable Systems Int., Las Vegas, NV, USA; henceforth: SSI). Measurements were made during the α-phase between 08:30 and 16:00 at *T*_a_’s ranging between ~ 22 and 44 °C. Air was drawn from outside the building using a compressor pump and stored in a tank, then dried and scrubbed of CO_2_ with an adsorption dryer (Ecodry K-MT 3, Parker Zander, Charlotte, NC, USA). Next, depending on the size of the group measured simultaneously, the air was continuously pushed through between eight and ten airtight 0.85 L respirometry chambers constructed of polypropylene containers (HPL 808, Lock&Lock, Hana Cobi, South Korea) placed in a temperature-controlled cabinet (ST-1200; Pol-Eko-Aparatura, Wodzisław Śląski, Poland). On a given day, each individual was measured at two randomly selected *T*_a_’s for ~ 3.5 h at each *T*_*a*_ which was sufficient to obtain post-absorptive values at the end of measurements. *T*_a_ in respirometry chambers was measured continuously with type-T thermocouples connected to two eight-channel readers (USB 4718; Advantech Europe, Munich, Germany) and was recorded on a PC with WaveScan software (ver. 2.0; Advantech Europe). The walls of each chamber were covered with black adhesive tape and equipped with a perch and metal mesh suspended ~ 4 cm above chamber floor in which a ~ 0.5 cm layer of mineral oil served as an excreta trap which prevented water evaporation. The main air flow was divided among the chambers, regulated at ~ 500 ml min^−1^ and measured upstream with two parallel mass-flow meter systems (Flow-Bar 4 and Flow Bar 8). At this flow rate, during measurements at highest *T*_a_’s, water vapor pressure in the chamber did not exceed 2.5 kPa (dewpoint < 21 °C). We used two parallel respirometry systems in which three and seven birds could be measured in parallel. In both systems, the excurrent airstream was subsampled at ~ 150 ml min^−1^ and pulled through a series of gas analyzers. Two computer-controlled multiplexers (MUX, SSI) automatically switched excurrent airstreams between animal chambers every 5 min. At least once every 20 min, the airstream was switched to a reference airline and the concentration of gases in the incurrent air was measured for 5 min. In the first system, partial pressure of water vapor (*P*_H2O_, kPa) in the airstream was measured with an RH-300 water vapor analyzer. Then, fractional concentrations of CO_2_ (FeCO_2_) and O_2_ (FeO_2_) in excurrent air stream were measured with a FoxBox-C integrated CO_2_ and O_2_ analyzer (SSI). In the second system *P*_H2O_, FeCO_2_ and FeO_2_ were analyzed in a sequence with Field Metabolic System analyzer (FMS; SSI). In both systems, air was dried with a Nafion™ drying tubes (product number 17049, VacuMed, Ventura, CA, USA) embedded in silica gel and then, in a column of magnesium perchlorate (anhydrous, ACS; Alfa Aesar GmbH & Co KG, Karlsruhe, Germany) before measuring FeCO_2_ and FeO_2_. The rate of O_2_ consumption (${\dot{V}} {{\rm O}}_{2}$) and CO_2_ production (${\dot{V}} {{\rm CO}}_{2}$; both in ml min^−1^) were calculated using Eqs. 10.6 and 10.7 of Lighton (2008). The rate of evaporative water loss (mg H_2_O min^−1^) was calculated using Eq. 10.9 of Lighton (2008) after verification for our system construction. Animal *T*_b_ was measured remotely with a PIT tag reader after completing measurement at each *T*_a_, when animals were still in the chambers. During whole-body and mask respirometry (see below), all elements of the measurement systems were controlled with a PC computer via an analog-to-digital interface (UI2, SSI) and data were acquired using ExpeData software (SSI) at 0.5 Hz.

### Mask respirometry

On completion of whole-body respirometry measurements, and after experimental acclimation of the birds, we analyzed the effect of acclimation on respiratory and cutaneous evaporative heat loss (REHL and CEHL, respectively). To do so, we measured respiratory and cutaneous evaporative water loss at *T*_a_’s of 25 and 40 °C, while the birds wore a mask (Online Resource Fig. 1; Tieleman and Williams 2002). Prior to measurement, birds were trained to wear a mask for a minimum of 30 min, at least 1 day before the trial. During measurement, a bird was placed in a 1.2 L glass chamber (Ikea 365 + , Ikea, Sweden) covered with a plastic lid lined with aluminum foil (to minimize H_2_O vapor adsorption on the plastic surface). Inside the chamber a wire mesh and a perch were placed ~ 2.5 cm above the bottom protected the animal from reaching a ~ 0.5 cm layer of mineral oil covering the bottom of the chamber. There was sufficient space for a bird to stand upright on the mesh during measurement. The bird’s head was secured with a rubber band in a polyethylene mask (Fig. 4, inset) which covered the whole head (thus REWL values included the H_2_O evaporation from the head skin and eye surfaces). Air flowed into the mask through the space between mask and head. Two small pumps pulled dry air from a column of silica gel into the chamber through a port protruding ~ 4 cm deep into the chamber. One pump pulled air through the mask (REWL line) at a constant rate of 500 ml min^−1^, while the second pump pulled air from the chamber (CEWL line) at 200 ml min^−1^. We used two separate systems to measure two birds at a time. In one system, air leaving the mask was pulled through an RH-300 (SSI) water vapor analyzer, dried with magnesium perchlorate, then passed through a mass-flowmeter and finally FeCO_2_ and FeO_2_ were measured with a FoxBox-C integrated CO_2_ and O_2_ analyzer (SSI). Air leaving the chamber was pulled through an RH-300 analyzer, dried with magnesium perchlorate, and the excurrent flow rate was measured with the mass-flowmeter of a subsampling pump (SS-4; SSI). In the second system, excurrent *P*_H2O_, FeCO_2_ and FeO_2_ were measured with Field Metabolic System analyzer, while in the chamber line, excurrent *P*_H2O_ was measured with RH-300 analyzer. In both lines of the second system, flow rates of air dried with magnesium perchlorate were measured with mass-flowmeters after measuring *P*_H2O_. Ambient temperature within chambers was measured continuously with calibrated thermistor probes (± 0.1 °C; 803-PS104R2, Mouser Electronics Inc., Mansfield, TX, USA)) attached to the lids of respirometry chambers. In both systems, $\dot{V}{\text{O}}_{2}$ and $\dot{V}{\text{CO}}_{2}$ were calculated using Eqs. 11.7 and 11.8 of Lighton (2008), while REWL and CEWL were calculated following Tieleman and Williams (2002). Respirometry chambers were placed in a temperature-controlled cabinet (Sanyo Incubator MIR-153, Sanyo Electronic Co. Ltd., Japan). Each measurement lasted 3 h, starting with a 1.5 h exposure to 25 °C. Then, temperature of the cabinet was set to 40 °C and the bird remained in the chamber for the following 1.5 h. *T*_a_ reached 40 °C after ~ 1 h, and the final recording lasted for ~ 0.5 h. Every 30 min a computer-controlled multiplexer (MUX; SSI) switched the airstream to a reference air that was sampled for 5 min. During that time, a separate pump attached to an outlet port of the solenoid valve of the multiplexer secured continuous flow of air through the mask and the chamber.

**Fig. 1:**
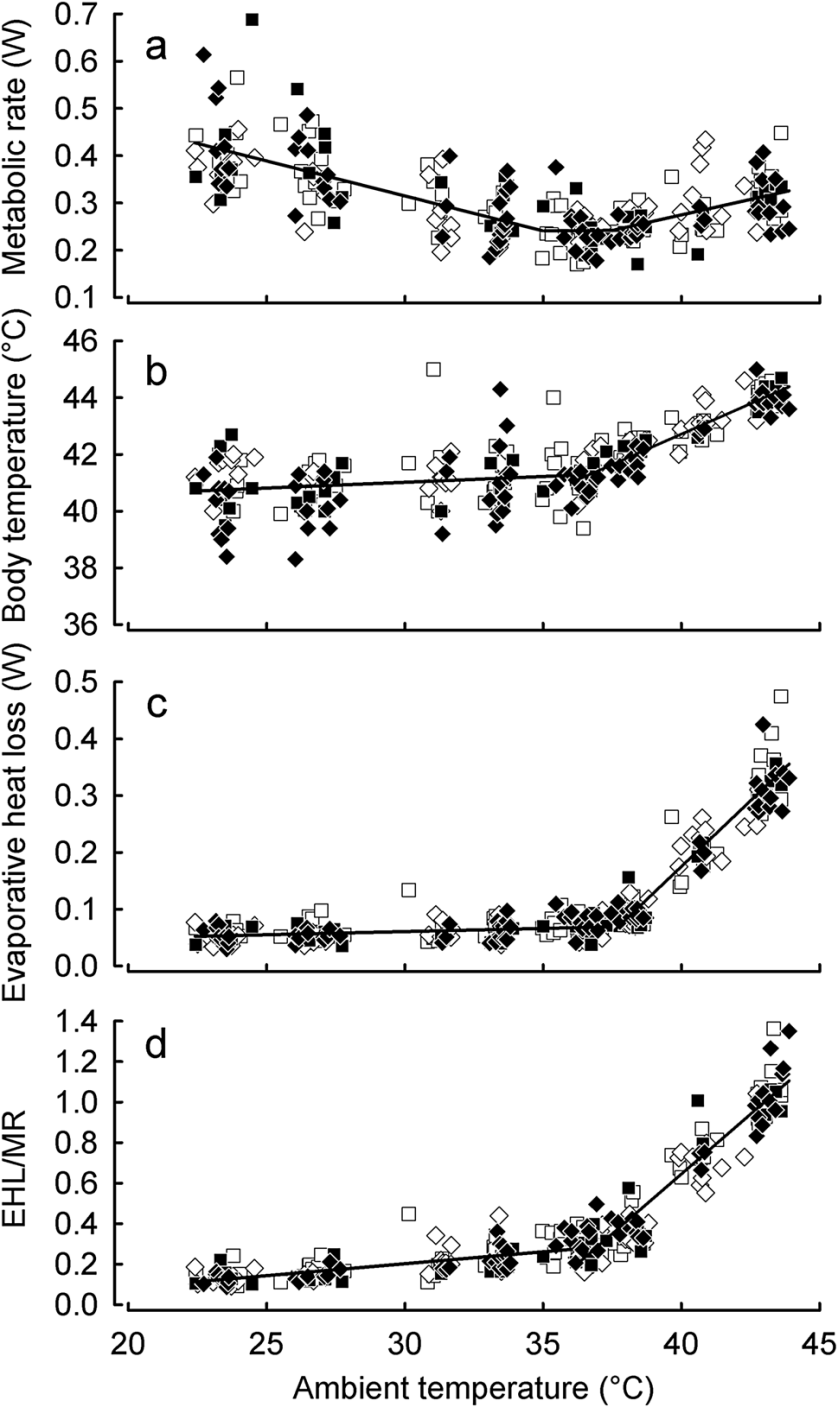
Metabolic rate (**a**), body temperature (**b**), evaporative heat loss (**c**) and efficiency of evaporative cooling (EHL/MR; **d**) as function of ambient temperature in zebra finches acclimated to *T*_a_ = 23 °C with unrestricted access to water. Data for initial acclimation are presented together as there was no difference between birds (GAMM: *p* > 0.1 for all variables). The solid line is the segmented linear regression model that provided the best fit. Different symbols stand for birds that were later assigned to different experimental acclimation

Bird *T*_b_ was measured at 30 s intervals and monitored continuously for the duration of the trial with the same system as during whole-body respirometry. In the case of excessive locomotor activity during measurement or excessive hyperthermia, the recording was terminated and the bird was immediately removed from the chamber. No fatalities occurred due to hyperthermia during or after measurements.

### Data analysis

Metabolic rate (MR, W) was calculated assuming a respiratory exchange ratio (RER, $\dot{V}{\text{CO}}_{2}/\dot{V}{\text{O}}_{2}$) calculated from recorded $\dot{V}{\text{CO}}_{2}$ and $\dot{V}{\text{O}}_{2}$ using oxyjoule equivalent calculated after Lighton et al. (1987):

$$\text{MR (W)}= \frac{\dot{V}{\text{O}}_{2}+5.164\cdot {\text{RER}}}{60}$$

Rate of evaporative heat loss (EHL, W) was calculated from EWL using a latent heat of vaporization of 2.4 J mg^−1^ H_2_O (Tracy et al. 2010), and the efficiency of evaporative cooling was calculated as the ratio EHL/MR. Thermal conductance (dry heat transfer coefficient, Tieleman and Williams 1999; C, W °C^−1^ cm^−2^) was calculated based on data collected below lower critical temperature (*T*_LC_, here: 34.87 ± 0.65 °C, see “Results” section) following Dawson and Schmidt-Nielsen (1966):

${\text{C }}({\text{W }}^\circ {\text{C}}^{{ - 1}} {\text{cm}}^{{ - 2}} ) = \frac{{{\text{MR}} - {\text{EHL}}}}{{({\text{T}}_{{\text{b}}} - {\text{T}}_{{\text{a}}} ) \cdot {\text{A}}_{{\text{s}}} }}$, where body surface area was calculated following Walsberg and King (1978) as *A*_s_ (cm^2^) = 10 *m*_b_^0.667^.

In the analysis of the respirometry data, we applied a steady-state approach and collected data were not corrected for instantaneous changes in gas concentrations (c.f. Bartholomew et al. 1981). MR and EHL during whole-body respirometry were calculated using 2 min averages of the lowest values of $\dot{V}{\text{O}}_{2}$ or concurrent value of EWL recorded at a given *T*_a_. For the mask respirometry, we used the average of the most unchanging continuous 5 min recording at a given *T*_a_. With that approach, we were able to select the lowest stable recording at 25 °C and the most stable recording during exposure to 40 °C. In both types of respirometry recordings, we selected *T*_b_ for the concurrent MR and EWL calculations.

Since MR, EHL and *T*_b_ did not differ between experimental groups after initial acclimation (generalized additive mixed effects models: *p* > 0.1, see “Results” section), we determined characteristic points of the Scholander-Irving model (Scholander et al. 1950) for all birds pooled together. After initial visual inspection of the relationships among MR, EHL and *T*_b_, and *T*_a_, we used SegReg software (www.waterlog.info/segreg.htm; Oosterbaan et al. 1990) to calculate segmented (piecewise) linear regression equations. In brief, the selection of a best fitting function describing the relationship and the breakpoint is done by maximizing the coefficient of determination and testing the significance of the model (Oosterbaan et al. 1990). Results of these analyses were used to determine *T*_LC_, upper critical temperature (*T*_UC_), the inflection point for EHL and the threshold *T*_a_ for hyperthermia; these values were presented ± S.E.

In the analysis of the effects of experimental acclimation on whole-body MR, EHL, *T*_b_ and efficiency of evaporative heat loss (EHL/MR), we used a two-step approach. First, to account for the curvilinear relationship between physiological variables of interest and *T*_a_, in the whole range at *T*_a_’s ranging between 22 and 44 °C (whole range of *T*_a_’s), we fitted generalized additive mixed effects models with the package “mgcv” ver. 1.8–31 (Wood 2006). These analyses allowed us to infer the effects of experimental acclimation on the whole animal response over the whole range of *T*_a_s to which birds were exposed. In all models, animal ID was set as a random factor to account for the repeated measurements of individuals. Initial models included acclimation (initial or experimental), acclimation regime (henceforth: group), and their interaction as fixed factors, *T*_a_ as a smoothed term and *m*_b_ as a covariate. Initial maximal models, including all fixed factors and interactions, were simplified by elimination of insignificant terms and the models were selected using information criteria (Crawley 2009). To meet the assumption of normal distribution of residuals (inspected visually), prior to analysis MR, EHL and EHL/MR were square-root transformed. Then, to infer the effect of experimental acclimation on MR, EHL, *T*_b_ and EHL/MR at the *T*_a_’s above the birds' upper critical temperature (*T*_UC_ = 37.47 ± 0.81 °C), we fitted linear mixed effects models (LME) to the data using “lme4” package ver. 1.1–23 (Bates et al. 2015). Initial maximal models included acclimation, experimental group, and their interaction as fixed factors, *T*_a_ and *m*_b_ as a covariates and animal ID as a random factor. Initial maximal models were simplified by stepwise elimination of insignificant terms (Crawley 2009). Prior to the analysis MR, EHL and EHL/MR were square-root transformed to follow the assumtions of linear modelling.

To test whether acclimation resulted in changes in heat loss at *T*_a_’s at which EHL is at its minimum, the thermal conductance was analyzed at *T*_a_’s below lower critical temperature using LME (lme4; Bates et al. 2015). Here, we included acclimation, group and their interaction as fixed factors, *T*_b_ and *T*_a_ as covariates and animal ID a random factor. Prior to analysis, C was square-root transformed. Since in all analyses of the whole-body variables we asked for the effect of acclimation, it was retained as a fixed factor in final models.

Partitioning of evaporative heat loss into cutaneous and respiratory avenues was analyzed by fitting LME to the cutaneous and respiratory EHL, ratio of REHL to CEHL (REHL/CEHL), and to ratio of CEHL to total EHL (CEHL/TEHL). To meet assumptions of linear modeling (Grafen and Hails 2002), all dependent variables were log-transformed. All initial maximal models included *T*_a_ at which measurement was taken (25 or 40 °C), acclimation group and their interaction as fixed factors. To account for repeated measurements of individuals in all models, animal ID was set as a random factor. In the model for REHL, MR and *m*_b_ were included as covariates. The model analyzing CEHL included *T*_b_ and *m*_b_ as covariates. In the analysis, initial maximal models were simplified by stepwise elimination of insignificant terms (Crawley 2009). Additionally, we analyzed whether MR, *T*_b_ and total (sum of respiratory and cutaneous) EHL differed between the measurements done using the whole-body and mask respirometry. We did so by fitting LME to the data collected by both methods at 25 and 40 °C with method of measurement and *T*_a_ (category) as fixed factors, and animal ID as a random factor. Both MR and EHL were log-transformed prior to analysis.

Repeatability (*τ*; Lessells and Boag 1987) of whole-body MR, EHL and *T*_b_ as well as CEHL and REHL was estimated for the final models with “rptR” ver. 0.9.22 (Stoffel et al. 2017). We also calculated repeatability of MR and total EHL measured at 25 and 40 °C using mask and whole-body respirometry. Data were presented as estimated marginal means ± SE which were calculated using “emmeans” package ver. 1.4.6 (Lenth 2020) and pairwise compared with Tukey’s HSD test adjusting for multiple comparisons. Marginal means for the whole range of *T*_a_’s were estimated and compared at the center of the *T*_a_ range (~ 34 °C), while for the analyses above the *T*_UC_ marginal means were estimated and compared at *T*_a_ = 44 °C. All above analyses were done in R ver. 4.0.0 (R Core Team 2020) Statistical significance was accepted at *p* < 0.05.

## Results

After initial acclimation, there was no difference between birds assigned to different acclimation groups in any of the variables analyzed (for the detailed results of the analyses see below). Thermoneutral zone ranged between *T*_LC_ = 34.87 ± 0.65 °C and *T*_UC_ = 37.47 ± 0.81 °C (Fig. 1a). Within TNZ, *m*_b_-adjusted BMR equaled 0.24 ± 0.0071 W. Below TNZ metabolic rate increased linearly by ~ 0.015 W °C^−1^, while above *T*_UC_ it increased at the rate of ~ 0.013 W °C^−1^. Up to *T*_a_ = 35.94 ± 0.14 °C zebra finches regulated their *T*_b_ at a relatively constant level of 40.98 ± 0.09 °C. Above this temperature, *T*_b_ increased linearly at the rate of 0.43 °C every degree of increase in *T*_a_ (Fig. 1b). This increase in *T*_b_ preceded an increase in EHL by ~ 1.5 °C. Below *T*_a_ = 37.45 ± 0.08 °C EHL was constant and averaged 0.061 ± 0.0014 W, and above this *T*_a_ it increased at a constant rate of 0.045 W °C^−1^ (Fig. 1c). Finally, below *T*_a_ = 36.16 ± 0.13 °C EHL accounted for dissipating ~ 20% of heat produced, yet above this *T*_a_, efficiency of evaporative heat loss (EHL/MR) increased steeply and at highest *T*_a_’s it equaled 1.4 (Fig. 1d).

### Whole-animal response to acclimation

**Fig. 2:**
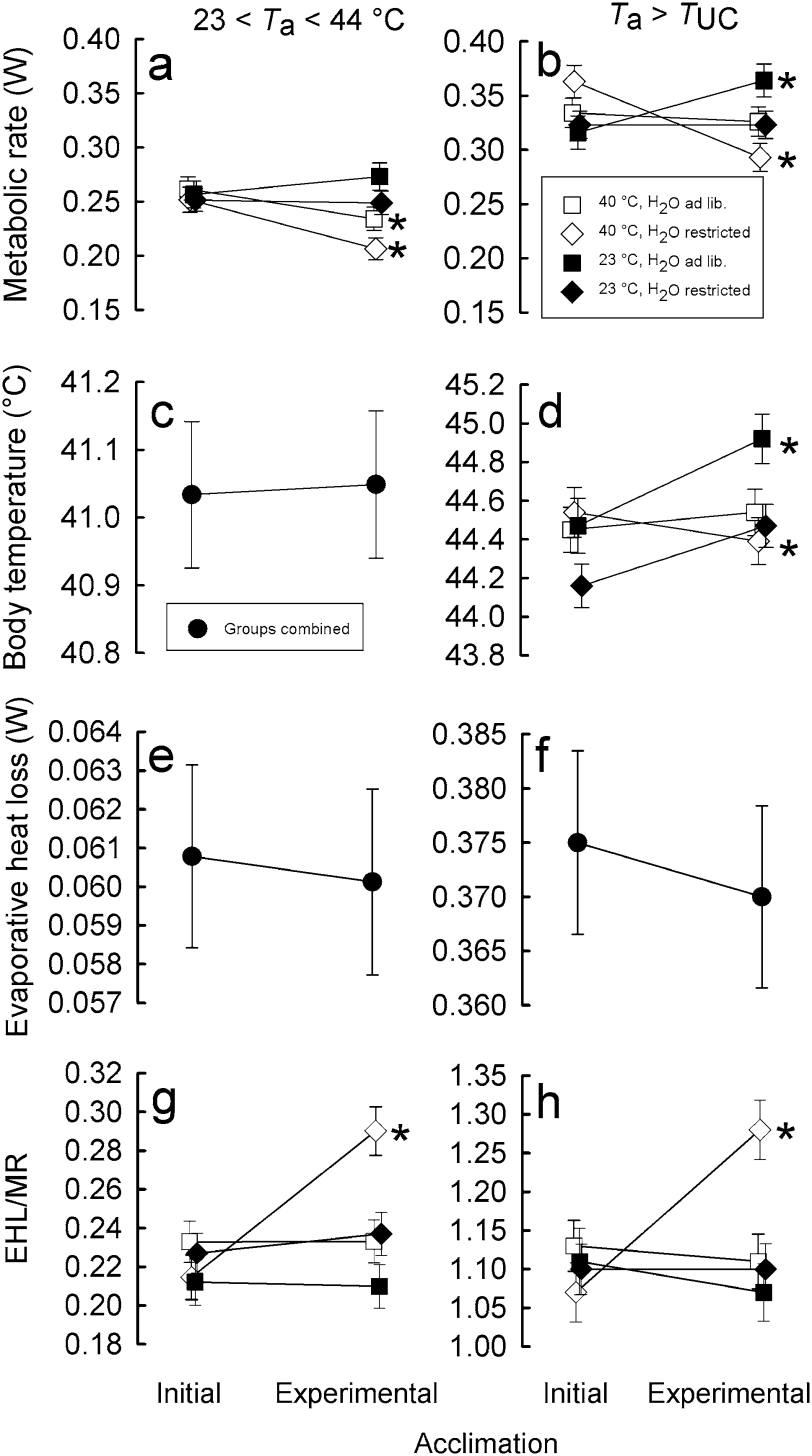
The effect of experimental acclimation on zebra finch metabolic rate (W; **a**, **b**), body temperature (°C; **c**, **d**), evaporative heat loss (W; **e**, **f**) and efficiency of evaporative heat loss (EHL/MR; **g**, **h**) at *T*_a_’s between 23 and 44 °C and above upper critical temperature (*T*_UC_). Different symbols (estimated marginal mean ± SE) stand for birds from different experimental groups. *T*_b_ (between 23 and 44 °C) as well as EHL (at all *T*_a_’s) of birds from different acclimation regimes did not differ (**c**, **e**, **f**) and thus groups were pooled together. Marginal means at *T*_a_ > *T*_UC_ were estimated at *T*_a_ = 44 °C. Asterisks indicate significant differences between measurements after initial and experimental acclimation (*p* < 0.0001)

Over the entire range of *T*_a_’s between 22 and 44 °C metabolic rate of zebra finches from all experimental groups were significantly correlated with *T*_a_ (*p* < 0.001) but not with *m*_b_ (*p* = 0.12). Changes in MR in response to experimental acclimation depended on the acclimation regime (interaction group × acclimation: *p* < 0.001, Table 1). After adjusting for *T*_a_ and *m*_b_, MR before experimental acclimation did not differ between groups (Tukey’s HSD: 0.89 < *p* < 1.00) and ranged between 0.25 ± 0.01 W and 0.26 ± 0.01 W (Fig. 2a). After adjusting for *m*_b_ and *T*_a_, mean MR of the group acclimated to 40 °C with H_2_O available *ad lib*. decreased by ~ 10% (*p* < 0.0001), and water restriction resulted in an additional decrease in MR of 10% (*p* < 0.0001). At the same time, MR of zebra finches acclimated to 23 °C, under both water regimes, did not differ from MR recorded after initial acclimation (Tukey’s HSD: *p* > 0.05).

**Table 1:** Results of the hypothesis tests for the generalized additive mixed models describing the relationship between ambient temperature (*T*_a_; smooth term), and zebra finch *Taeniopygia guttata* response variables (metabolic rate [MR], body temperature [*T*_b_], evaporative heat loss [EHL] and efficiency of evaporative heat loss [EHL/MR]) after adjusting for body mass, acclimation (Acl) and acclimation regime (group)

Response variable	Smooth term (*T*_a_)			Parametric terms			
	e.d.f	*F*	*p*	Factor	*df*	*F*	*p*
Sqrt MR	**6.37**	**117.9**	** < 0.0001**	*m*_b_	1	2.425	0.12
				group	3	2.183	0.089
				**Acl**	**1**	**16.020**	** < 0.0001**
				**group × Acl**	**3**	**12.559**	** < 0.0001**
*T*_b_	**6.037**	**207.6**	** < 0.0001**	***m***_**b**_	**1**	**13.307**	**0.00029**
				Acl	1	0.052	0.82
sqrt EHL	**7.69**	**700.3**	** < 0.0001**	***m***_**b**_	**1**	**15.176**	**0.0001**
				Acl	1	0.194	0.66
sqrt (EHL/MR)	**7.17**	**713.7**	** < 0.0001**	*m*_b_	1	1.591	0.21
				**group**	**3**	**3.940**	**0.0080**
				**Acl**	**1**	**13.264**	**0.00030**
				**group × Acl**	**3**	**10.531**	** < 0.0001**

Smooth term results show the ANOVA results for the effect of *T*_a_, and parametric terms show results for *m*_b_ (covariate), group, acclimation and their interaction (fixed factors). Bold font indicates significant relationships (*p* < 0.05)

*E.d.f*. estimated degrees of freedom

Changes in whole-animal metabolic heat production at *T*_a_’s above the birds' *T*_UC_, after adjusting for *m*_b_ (*F*(1, 49.4) = 6.72, *p* < 0.05) and *T*_a_ (*F*(1, 127.8) = 119.77, *p* < 0.0001), differed between acclimation regimes (interaction group × acclimation: *F*(3, 131.1) = 14.42, *p* < 0.0001). Water-restricted birds acclimated to 40 °C decreased MR by ~ 20% (*p* < 0.0001) compared to initial acclimation, while MR in the birds with unlimited access to H_2_O as well as individuals acclimated to 23 °C but with restricted H_2_O did not change at *T*_a_’s above *T*_UC_ (*p* > 0.05). Finally, MR in zebra finches kept continuously under the initial conditions (23 °C throughout the day and H_2_O *ad lib.*) increased at high *T*_a_’s by ~ 15% (*p* < 0.01; Fig. 2b). Metabolic rate measured at *T*_a_’s above *T*_UC_ differed consistently among individuals when adjusted for *T*_a_, *m*_b_, group and acclimation (*τ* = 0.39 ± 0.09, *p* < 0.0001).

When analyzed over the entire range of *T*_a_’s, variations in body temperature correlated with *T*_a_ (*p* < 0.0001) and *m*_b_ (*p* < 0.001, Table 1) and did not differ between acclimation regimes (*p* = 0.82; Fig. 2c). Since *T*_b_ did not differ between experimental groups, both before and after experimental acclimation, this term was dropped from the final model. After adjusting for *T*_a_ and *m*_b_, before experimental acclimation, *T*_b_ ranged between 41.03 ± 0.11 °C and 41.05 ± 0.11 °C (Fig. 2c).

After acclimation to initial conditions zebra finches began to develop hyperthermia at *T*_a_’s >  ~ 36 °C (see above). After acclimation to 40 °C in birds with unrestricted access to water hyperthermia started to develop at *T*_a_’s > 32.72 ± 0.37 °C, like in birds acclimated to 23 °C with restricted water availability (32.92 ± 0.10 °C). In the remaining groups, we did not observe changes in threshold for initiation of hyperthermia.

At *T*_a_’s above the birds' upper critical temperature, *T*_b_ increased with increasing *T*_a_ (*F*(1, 129.0) = 799.00, *p* < 0.0001) by ~ 0.4 °C every 1 °C. When exposed to high *T*_a_’s during metabolic measurements, birds acclimated to constant *T*_a_ = 23 °C, both with water *ad lib.* or restricted, reached *T*_b_’s higher by ~ 0.4 °C than during measurements at the same *T*_a_’s after initial acclimation (significant interaction group × acclimation: *F*(3, 131.8) = 4.97, *p* < 0.01, Fig. 2d). When birds acclimated to 40 °C were measured at high *T*_a_’s, their *T*_b_ did not differ between acclimation regimes (*p* > 0.05 for both hydric regimes, Fig. 2d). Repeatability of *T*_b_ measured during respirometry trials at *T*_a_’s above *T*_UC_ equaled 0.29 ± 0.09 (*p* < 0.0001).

Evaporative heat loss changed neither in response to different thermal conditions nor H_2_O availability (*p* = 0.67, Fig. 2e). Over the whole range of *T*_a_’s, variations in EHL were explained only by *m*_b_ (*p* < 0.001) and *T*_a_ (*p* < 0.0001, Table 1). Above *T*_UC_, irrespective of group or acclimation, the rate of evaporative heat loss correlated only with *m*_b_ (*F*(1, 54.9) = 9.66, *p* < 0.01) and *T*_a_ (*F*(1, 139.3) = 1025.30, *p* < 0.0001). After adjusting for *T*_a_, *m*_b_ and acclimation regime EHL differed consistently among individuals (*τ* = 0.15 ± 0.08, *p* < 0.01).

Over the entire range of *T*_a_'s at which MR was measured, the ratio of EHL to MR was positively related to *T*_a_ (*p* < 0.0001), but not to *m*_b_ (*p* > 0.05, Table 1). The efficiency of evaporative heat loss changed in response to experimental acclimation, and the magnitude of this change differed among acclimation treatments (interaction group × acclimation: *p* < 0.0001, Table 1). After adjusting for *T*_a_ and *m*_b_ initial values of EHL/MR did not differ among experimental groups (0.43 < *p* < 1.00) and ranged between 0.21 ± 0.01 and 0.23 ± 0.01 (Fig. 2g). After acclimation to 40 °C with water restriction, efficiency of EHL increased by 38% compared to initial conditions (0.29 ± 0.13; Tukey’s HSD: *p* < 0.0001), while in birds from remaining groups, it did not change (Fig. 2g).

The results were very similar when changes in efficiency of EHL at high *T*_a_’s were analyzed. It increased with increasing *T*_a_ (*F*(1, 135.2) = 917.86) and did not correlate with *m*_b_ (*p* > 0.05). When compared at 44 °C, experimental acclimation resulted in ~ 20% increase in EHL/MR only in water-restricted birds acclimated to 40 °C during daytime (*p* < 0.0001), while in other groups, the effect of acclimation was not detectable (*p* > 0.05; interaction group × acclimation: *F*(3, 136.3) = 6.93, *p* < 0.001, Fig. 2h).

**Fig. 3:**
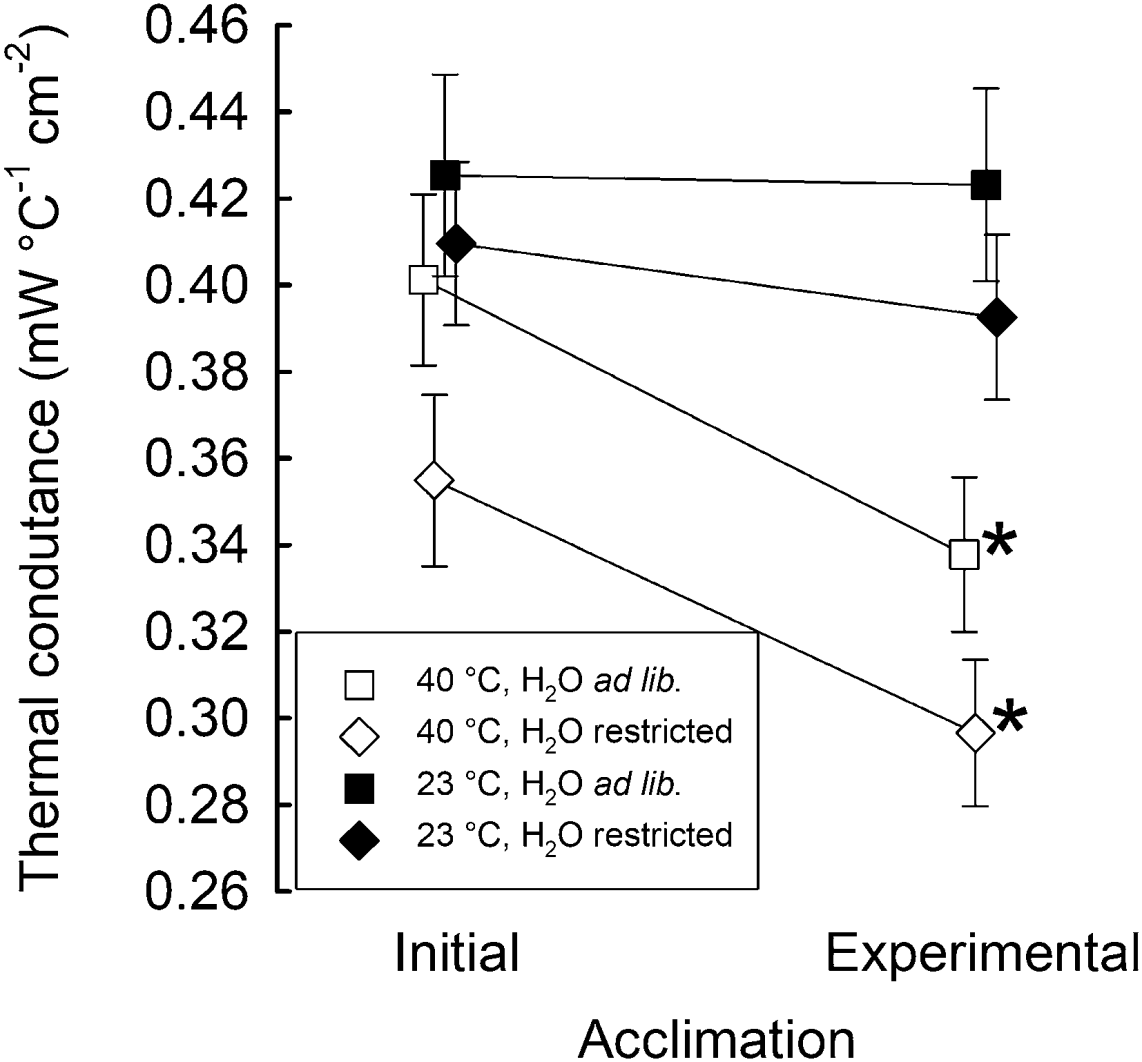
Thermal conductance (mW °C^−1^ cm^−2^; estimated marginal mean ± SE) below lower critical temperature after initial and experimental acclimation. Different symbols stand for zebra finches from different acclimation regimes. Asterisks indicate significant differences within groups (*p* < 0.05)

Overall, at *T*_a_’s below lower critical temperature, the thermal conductance was affected by *T*_a_ (*F*(1, 199.0) = 79.78, *p* < 0.0001) and *T*_b_ (*F*(1, 217.8) = 12.25, *p* < 0.001), and its change due to acclimation was affected by acclimation regime (interaction group × acclimation: *F*(3, 196.7) = 3.10, *p* < 0.05). In birds acclimated to 40 °C conductance decreased with acclimation by ~ 16% (*p* < 0.001), irrespective of water availability. At the same time, acclimation to 23 °C throughout the day did not affect C, irrespective of water availability (Fig. 3).

### Partitioning of evaporative heat loss

After accounting for the effect of ambient temperature (25 and 40 °C), the sum of respiratory and cutaneous heat loss measured in birds wearing a mask was ~ 70% higher than evaporative heat loss measured in unrestrained birds during whole-body measurements (Online Resource Fig. 2a, b). At the same time, metabolic rate measured with a mask system exceeded MR of unrestrained birds by ~ 33% (Online Resource Fig. 2c, d), while *T*_b_ did not differ during the two measurement methods (Online Resource Fig. 2e, f). Nevertheless, EHL, MR and *T*_b_ were repeatable after accounting for different measurement methods and ambient temperature (EHL: *τ* = 0.20 ± 0.10, *p* < 0.05, MR: *τ* = 0.22 ± 0.10, *p* < 0.01, and *T*_b_: *τ* = 0.18 ± 0.10, *p* < 0.05).

**Fig. 4:**
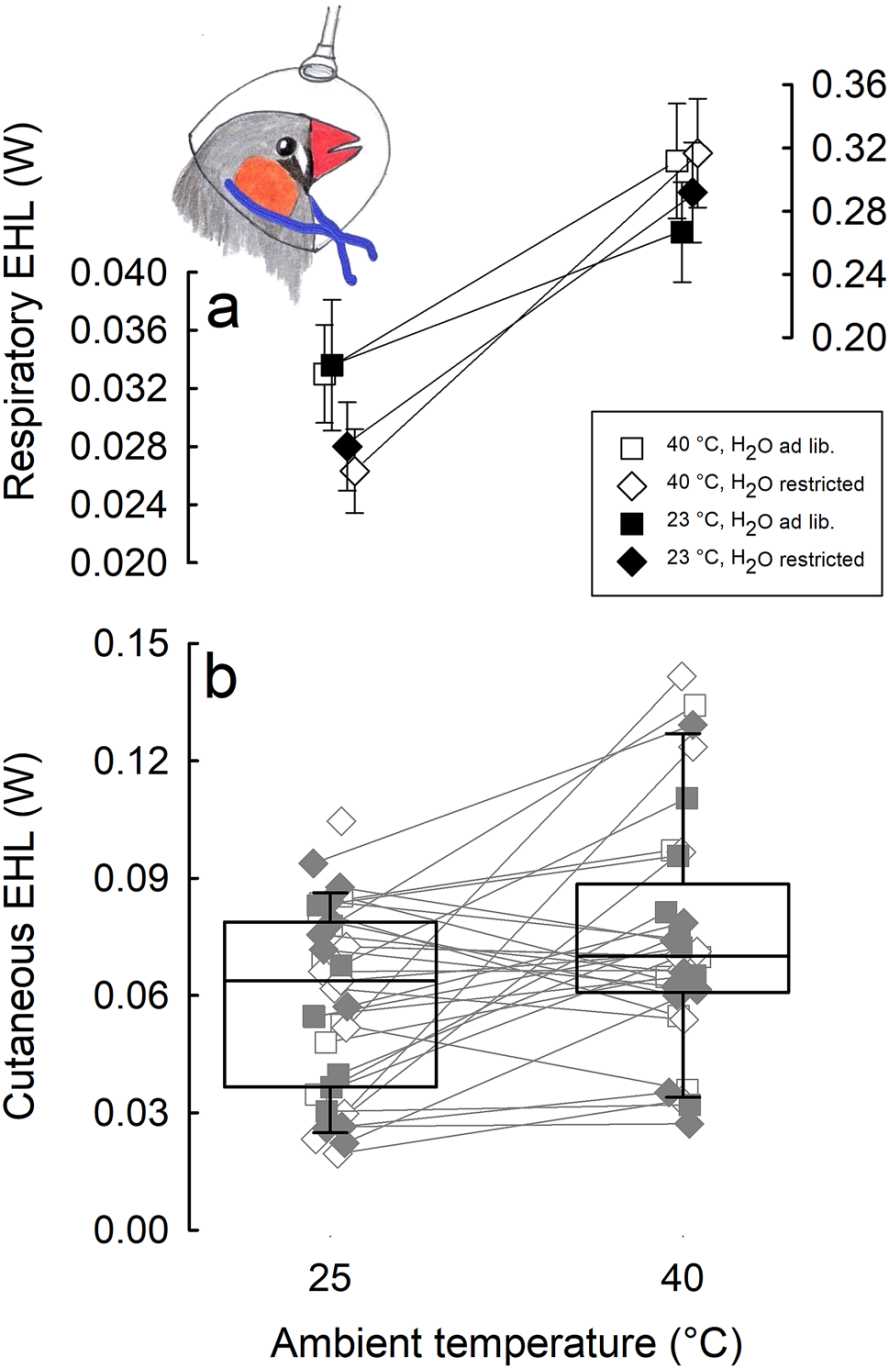
Respiratory (**a**) and cutaneous (**b**) evaporative heat loss (W; estimated marginal mean ± SE) at 25 and 40 °C in zebra finches after experimental acclimation. Different symbols stand for birds from different acclimation regimes. Note different scale of axes for parts at 25 and 40 °C at panel of respiratory EHL (**a**). Boxes at panel of cutaneous EHL (**b**) indicate 25th and 75th percentiles, solid line stands for median. Different symbols mark individual birds from different acclimation regimes. Inset: a schematic drawing of zebra finch in the polyethylene mask used for respiratory evaporative water loss measurements. Rubber band securing the mask (blue) was placed under feathers below the occipital region of the skull (color figure online)

Respiratory evaporative heat loss was positively correlated with MR (*F*(1, 48.7) = 54.52, *p* < 0.0001) and was higher at 40 than at 25 °C, but the magnitude of this difference depended on acclimation regime (interaction group × *T*_a_: *F*(3, 30.3) = 3.08, *p* < 0.05). Namely, the difference between 25 and 40 °C was slightly greater in water-restricted birds under both thermal regimes than in birds having unlimited access to water (Fig. 4a). Individual REHL was highly and significantly repeatable between 25 and 40 °C when adjusted for *m*_b_, MR and acclimation regime (*τ* = 0.60 ± 0.11, *p* < 0.0001).

Cutaneous EHL loss did not correlate with *T*_b_, MR, *m*_b_ or acclimation regime and was higher by ~ 25% when birds were exposed to 40 than to 25 °C (*F*(1, 33.0) = 8.79, *p* < 0.01, Fig. 4b). After adjusting for *T*_a_, CEHL showed consistent among individual differences (*τ* = 0.41 ± 0.14, *p* < 0.01).

**Fig. 5:**
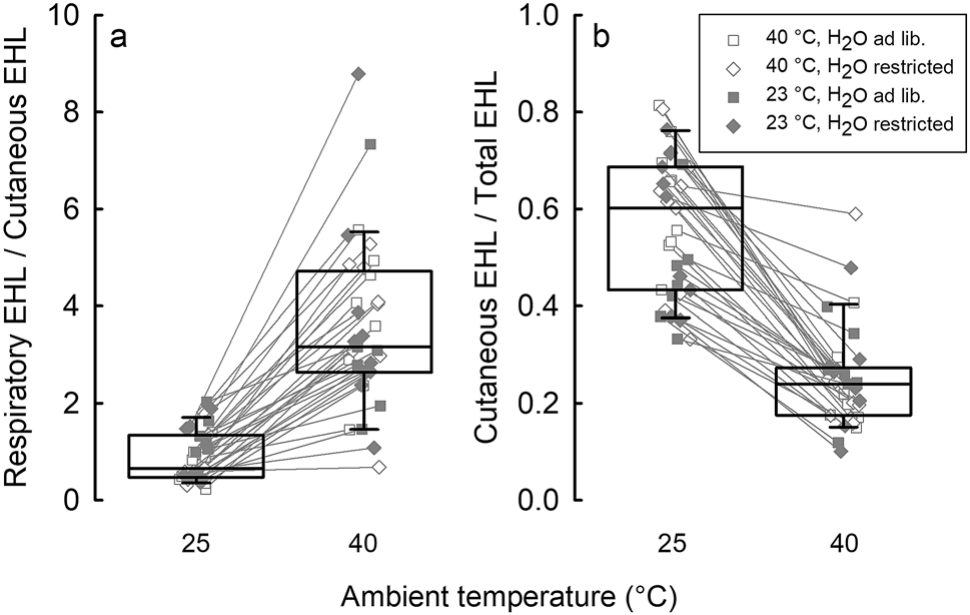
Respiratory to cutaneous evaporative heat loss (EHL) ratio (**a**), and contribution of cutaneous EHL to total EHL (**b**) at *T*_a_ of 25 and 40 °C. Boxes indicate 25th and 75th percentiles, solid lines stand for median. Different symbols indicate individual birds from different acclimation regimes

There was no difference between birds acclimated to different thermal and water regimes in the REHL/CEHL (*p* > 0.05), but this ratio differed between 25 and 40 °C (*F*(1, 33.0) = 173.56, *p* < 0.0001). At *T*_a_ = 25 °C, cutaneous evaporative heat loss was over twofold higher than respiratory EHL, while at *T*_a_ = 40 °C, the ratio reversed and REHL exceeded CEHL over threefold (Fig. 5a). As indicated by the analysis of CEHL/TEHL, after adjusting for MR (*F*(1, 55.6) = 5.81, *p* < 0.05), cutaneous avenue at 25 °C accounted for 61% of the total EHL, whereas at 40 °C, its contribution decreased to 21% (*F*(1, 48.2) = 104.74, *p* < 0.0001, Fig. 5b).

## Discussion

Zebra finches, a small arid zone passerine, increased the efficiency of evaporative heat loss (Fig. 2g, h) and thus precision of *T*_b_ regulation (Fig. 2d) in response to prolonged exposure to hot and desiccating conditions. Being more effective under harsher conditions, efficiency of EHL was facilitated by a decrease in metabolic heat production (Fig. 2a, b) as the main response in our experimental study. Contrary to our predictions, birds acclimated to high *T*_a_ or to water restriction, or both did not decrease total evaporative heat loss (Fig. 2e, f). Yet, irrespective of acclimation temperature, water restriction resulted in lowering of respiratory evaporative heat loss at *T*_a_ below thermoneutrality (Fig. 4a). Consistent among-individual differences (repeatability) in metabolic rate, body temperature, and in total, respiratory and cutaneous water loss suggest that these traits may be a target for selection favoring phenotypes most effectively responding to thermally challenging conditions.

### Whole-animal response to acclimation

Before acclimation to experimental conditions, mean zebra finch BMR equaled 0.24 W which agrees with values reported previously for the species (Cade et al. 1965; Calder 1964) and other similar-sized passerines (McKechnie and Swanson 2010; McKechnie and Wolf 2004a). The differences in MR outside the thermoneutral zone between our results and values reported earlier (Calder 1964; Cooper et al. 2020b) may result from the differences in time of the day when MR was measured. Namely, our birds were measured during α-phase, Cooper et al. (2020b) measured birds during ρ-phase, and birds of Calder (1964) were kept under an inverted light–dark cycle. In passerines, heat loss considerably differs between α and ρ-phases, being ~ 50% lower in ρ-phase (Aschoff 1981). Lower critical temperature recorded in present study corresponds well to the value reported by Cade et al. (1965), but is higher by ~ 6 °C than that reported by Calder (1964), while upper critical temperature (*T*_UC_) is ~ 2.5 °C lower than reported by Calder (1964) and Cade et al. (1965). The most plausible explanation for this difference was ~ 20% lower MR of birds at *T*_a_’s between 42 and 44 °C in our study than in Calder (1964).

Our results concur with most recent studies on thermoregulatory responses of zebra finches to simulated and natural heat waves (Cooper et al. 2020a, b). The same shape of relationship between MR and *T*_a_ recorded after initial and experimental acclimation (Fig. 1) suggests no biologically meaningful change in the lower and upper critical temperatures in response to acclimation to heat, as it was suggested by Cooper et al. (2020a). The main response of zebra finches to 40 °C during daytime was the overall decrease of heat production over the entire range of ambient temperatures, which was further augmented by water restriction (Fig. 2a, b). Flexible adjustment of MR to prevailing thermal conditions reflects a common reaction of small endothermic homeotherms to changes in energy demands (for reviews see: Bicudo et al. 2010; McKechnie 2008; Piersma and Van Gils 2011). Acclimation to *T*_a_’s below thermoneutrality lead to increased BMR (Barcelo et al. 2017; Vezina et al. 2017), while acclimation to *T*_a_’s > *T*_LC_ resulted in MR decrease both in birds (Harrison and Biellier 1969; McKechnie et al. 2007; Noakes and McKechnie 2020; Tieleman et al. 2003; Vezina et al. 2006; Williams and Tieleman 2005, 2000) and in mammals (Boratyński et al. 2016b; Chaffee and Roberts 1971; Nespolo et al. 2001). However, at *T*_a_’s above thermoneutrality, only zebra finches acclimated to high *T*_a_ and restricted water availability reduced metabolic heat production. This suggests that the overall reduction of MR in the groups acclimated to high diurnal *T*_a_ (Fig. 2a) was due to reduced metabolism at *T*_a_’s below their *T*_UC_. Our results suggest that heat-acclimated birds exposed to *T*_a_’s below their *T*_UC_ may conserve energy, reduce the use of energy reserves and even accumulate it when in positive energy balance. Eventually, when exposed to high *T*_a_’s with limited water availability, birds could use these body reserves as a source of water produced in fat and protein catabolism. Support for this mechanism comes from studies by Gerson and Guglielmo (2011), Rutkowska et al. (2016) and McCue et al. (2017) who showed that both birds and mammals may rely on fat and protein catabolism to sustain water needs during dehydration, fasting and sustained exercise during migration flight.

Overall, regulation of body temperature in zebra finches below their *T*_LC_ was not affected by acclimation regimes. At *T*_a_ > 36 °C, *T*_b_ increased linearly together with linear increase of evaporative heat loss and the efficiency of evaporative heat loss (EHL/MR), as in other Australian and Southern African passerines of similar *m*_b_ (Freeman et al. 2020; McKechnie et al. 2017; Whitfield et al. 2015). Conversely, Cooper et al. (2020b) found that acclimation of zebra finches to experimental conditions mimicking heat-wave did not increase their *T*_b_ as it was recorded under natural conditions (Cooper et al. 2020a). But they measured *T*_b_ only at 30 and 40 °C. In our study, after initial acclimation, all birds started to develop hyperthermia at *T*_a_ of ~ 36 °C, i.e. ~ 1.5 °C lower than their *T*_UC_ and lower than temperature at which evaporative heat loss started to increase (Fig. 1). Regulated hyperthermia is one of the main mechanisms allowing birds to minimize water loss at high *T*_a_’s (Calder and King 1974; Gerson et al. 2019; Tieleman and Williams 1999). One might expect that acclimation to high *T*_a_ and water restriction would lead to a shift of the hyperthermia threshold to lower *T*_a_’s. This was partially true. Only birds acclimated to 40 °C with ad libitum access to water, and birds acclimated to 23 °C with water restriction, initiated hyperthermia at *T*_a_’s lower by ~ 3 °C than before experimental acclimation. Despite these differences, we found a significant effect of acclimation regime on *T*_b_ at *T*_a_’s above the birds' *T*_UC_ (Fig. 2d). Birds acclimated to 40 °C irrespective of water availability regulated *T*_b_ at the same level as before acclimation, while zebra finches acclimated to 23 °C increased it significantly. On the one hand, this may indicate that heat-acclimated individuals dissipated heat more effectively. This might be true for birds acclimated to heat and restricted water (Fig. 2g, h). On the other hand, thermal conductance in birds acclimated to 40 °C decreased by ~ 16%, irrespective of water regime, while in birds acclimated to 23 °C, it did not change (Fig. 3). We also cannot exclude the possibility of additional changes in heat transfer resulting from e.g. vasomotor changes during exposure to high *T*_a_’s in hot acclimated birds. As suggested by Dawson and Schmidt-Nielsen (1966), at high *T*_a_’s, endothermic animals can prevent heat flow from the environment to the body by decreasing thermal conductance. Also, summer acclimation may result in decreased dry heat transfer at ambient temperatures exceeding *T*_b_ (Tieleman et al. 2002b; but see: Tieleman and Williams 1999) possibly due to vasoconstriction of the peripheral blood vessels. A few studies of desert passerines show contradictory results. On one hand, white-browed sparrow-weavers *Plocepasser mahali* acclimated to day-time temperatures between 30 and 42 °C increased the threshold for hyperthermia in response to acclimation to high *T*_a_ (Noakes and McKechnie 2019). At the same time, birds acclimated to 40 °C maintained lower *T*_b_ than those acclimated to 30 °C (Noakes and McKechnie 2019). On the other hand, another study found no effect of high *T*_a_ on *T*_b_ (Oswald et al. 2018) and reported that acclimatization to hot summers resulted in lower *T*_b_ when birds were exposed to *T*_a_’s > 40 °C (Noakes et al. 2016). Yet, both Noakes et al. (2016) and Oswald et al. (2018) found that acclimatization to summer was accompanied by significant increases in EWL as well as in the efficiency of evaporative cooling. Noakes et al. (2016) reported that maintenance of lower *T*_b_ at high *T*_a_’s by white-browed sparrow-weavers in summer correlated with seasonally lower MR, which was not observed in Cape rockjumpers *Chaetops frenatus* (Oswald et al. 2018). Comparison of our results with the above studies shows that acclimation or acclimatization to hot and desiccating conditions in desert adapted species leads to improved thermoregulation at high *T*_a_’s. This seems to be achieved by changes in physiological mechanisms improving the efficiency of evaporative cooling, both by changes in evaporative heat loss or metabolic heat production or both, which additionally may be facilitated by adjustments in thermal conductance. However, it seems that all of these adjustments need to be accompanied by high tolerance of dehydration as is often characteristic for desert-adapted species (Maclean 1996). Since metabolic water production is directly related to metabolic heat production, lower metabolic rate in hot acclimated and water-restricted birds should result in lower rate of water release. Eventually, lack of changes in whole-body EHL in zebra finches or increased evaporative water loss in other species would lead to inevitably higher water loss. Without evolving mechanism of increased dehydration tolerance, the observed responses to acclimation or acclimatization to hot and desiccating conditions would need to be selected out by natural selection.

### Partitioning of evaporative heat loss

In this study, we also asked whether prolonged exposure to high temperature and water restriction affected partitioning of evaporative heat loss between respiratory and cutaneous avenues. The sum of respiratory and cutaneous EHL measured with mask respirometry considerably excided EHL measured with whole-body approach (see “Results” section and Online Resource Fig. 2). This difference may result from two reasons. We have no doubt that stress resulting from immobilization and wearing a mask lead to increased metabolic rate (and thus increased respiration rate; however, it was rather not associated with stress hyperthermia or emotional fever since *T*_b_ did not differ between the two measurement methods) compared to unrestrained individuals leading to greater water loss from the respiratory evaporative surfaces. Second, the high flowrate of air through the mask could result in faster depletion of humid air leading to greater heat loss in finches wearing a mask (c.f. Gerson et al. 2014). Lack of differences in *T*_b_ of masked and unrestrained birds supports this possibility. Nevertheless, the results obtained with both methods, although quantitatively different, were repeatable allowing us to conclude about the effect of experimental acclimation on evaporative heat loss partitioning in zebra finches.

**Fig. 6:**
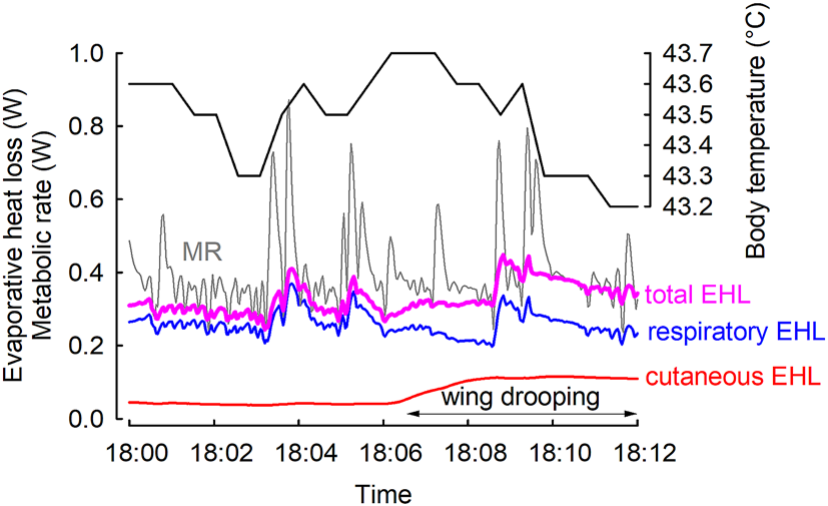
Representative recording of body temperature (black line), metabolic rate (MR, grey line), total evaporative heat loss (total EHL, REHL + CEHL; pink line), respiratory evaporative heat loss (blue line), and cutaneous evaporative heat loss (red line) in one zebra finch measured at *T*_a_ = 40 °C. Black arrows indicate the time of wing drooping (color figure online)

Since water deprivation was found to affect both total (Calder 1964; Greenwald et al. 1967) and cutaneous evaporation (Arad et al. 1987), we expected that acclimation to water restriction would result in lower cutaneous evaporative heat loss than in birds having unlimited access to water. Similarly, acclimation to desiccating conditions in house sparrows *Passer domesticus* resulted in reduction of cutaneous water loss (Muñoz-Garcia et al. 2008). However, this was not the case in present study. Overall, changes in EHL partitioning with *T*_a_ (Figs. 4 and 5) agree with patterns observed in other passerine species (Tieleman and Williams 2002; Wolf and Walsberg 1996). Interestingly, in water-restricted birds acclimated to both thermal regimes, we observed a slightly lower respiratory EHL at 25 °C than in birds having unrestricted access to water (Fig. 4a). Lower REHL at 25 °C might have provided water savings necessary for thermoregulation at high *T*_a_’s. The underlying mechanism may be related to differences in blood flow to the walls of respiratory tract or changes in counter-current heat exchange in cranial circulation (Bernstein 1982). Although CEHL did not contribute much to total evaporative heat loss at 40 °C (Fig. 5), our observations suggest that it may be of great importance for birds exposed to high *T*_a_’s. On several occasions, we observed that during measurements at 40 °C CEHL increased periodically by more than 50% and this change was accompanied by subsequent decrease of *T*_b_. Examination of the heat loss and metabolic heat production indicated that this change in CEHL resulted in increase of TEHL to a level comparable to metabolic heat production (Fig. 6). Increase in CEHL was also accompanied by wing drooping which exposed underwing apteria. Unfortunately, these were only accidental observations which did not permit formal analysis of this behavior. Yet, it is well documented that wing drooping is one of the most common behaviors in hot exposed birds (Pattinson et al. 2020; Smit et al. 2016) and together with our observations may suggest that it may be responsible for an important change in total heat loss, together with gular fluttering and panting at a resonant frequencies of respiratory system (c.f. McKechnie and Wolf 2019).

## Conclusions

Zebra finches exposed to hot and desiccating conditions flexibly adjusted metabolic heat production which increased the efficiency of evaporative heat loss and facilitated regulation of body temperature. Such phenotypic flexibility may be crucial in response to changes in environmental conditions, especially in the face of present and predicted climate change (present study; Cooper et al. 2020b; Noakes and McKechnie 2019; Noakes et al. 2016; Oswald et al. 2018; Tieleman et al. 2002b, 2002a; Williams and Tieleman 2000, 2002). Possibly, this phenotypic flexibility may also undergo seasonal changes as it was shown in mammals (Boratyński et al. 2016a, 2017b). Also, since thermal history may affect flexibility of energy metabolism (Barcelo et al. 2009), we cannot exclude that acclimation to high *T*_a_’s in summer may additionally facilitate flexible adjustments of thermoregulatory traits. This would be of great importance especially since probability of extreme weather events is increasing with increasing mean summer *T*_a_ (Conradie et al. 2019; Harris et al. 2018). However, even though potential benefits of flexible adjustments in physiology improve heat tolerance in birds, both theoretical predictions as well as empirical data show population declines in many species living in hot and dry regions of the globe (Albright et al. 2017; Conradie et al. 2019, 2020; McKechnie and Wolf 2010; Riddell et al. 2019). Significant repeatability of whole animal metabolic rate, total evaporative heat loss, as well as cutaneous and respiratory heat loss suggest that these traits may be a subject to natural selection, provided that they are heritable (Boratyński et al. 2017a; Dohm 2002; Rønning et al. 2005, 2007). Current and future selection pressure may favour individuals with the greatest potential for phenotypic changes in heat tolerance (c.f. Cooper et al. 2020b; McKechnie et al. 2012) resulting in greater tolerance of short-term extreme weather events.

## Electronic supplementary material

Below is the link to the electronic supplementary material.

Supplementary file1 (PDF 245 kb)

## Data Availability

The datasets generated during and/or analysed during the current study are available from the corresponding author on a reasonable request.

## Code availability

Code will be available upon request to the authors.
