# Supplementary material for: Phenotypic flexibility in heat production and heat loss in response to thermal and hydric acclimation in the zebra finch, a small arid-zone passerine
Source: J Comp Physiol B. 2020 Oct 18;191(1):225–39. doi: 10.1007/s00360-020-01322-0 (PMC7819915; doi:10.1007/s00360-020-01322-0)
Supplement: Supplementary file 1 — Supplementary file1 (PDF 245 kb) [file 360_2020_1322_MOESM1_ESM.pdf]

Online resource

Journal of comparative physiology B

Phenotypic flexibility in heat production and heat loss in response to thermal and hydric acclimation in the zebra finch, a small arid-zone passerine

Michał S. Wojciechowski[1,*], Anna Kowalczewska[1], Roger Colominas-Ciuró[1], Małgorzata Jefimow[2]

1. Department of Vertebrate Zoology and Ecology, Faculty of Biological and Veterinary Sciences, Nicolaus Copernicus University, Toruń, Poland.

2. Department of Animal Physiology and Neurobiology, Faculty of Biological and Veterinary Sciences, Nicolaus Copernicus University, Toruń, Poland.

*corresponding author email: mwojc@umk.pl

ORCID: 0000-0001-7765-0720

Figure 1

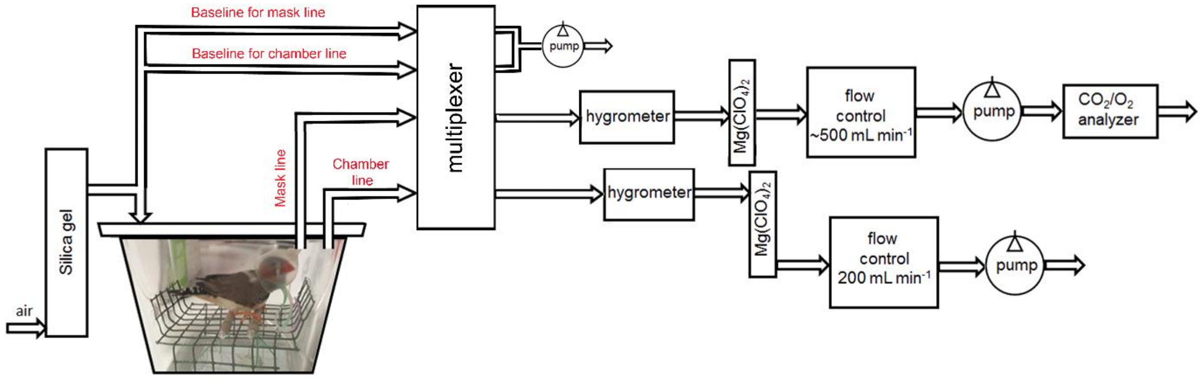

Fig. 1 Diagram of the respirometry system used in the measurements of respiratory (mask line) and cutaneous (chamber line) evaporative water loss in zebra finches *Taeniopygia guttata*. See text for the detailed description of the system

Figure 2

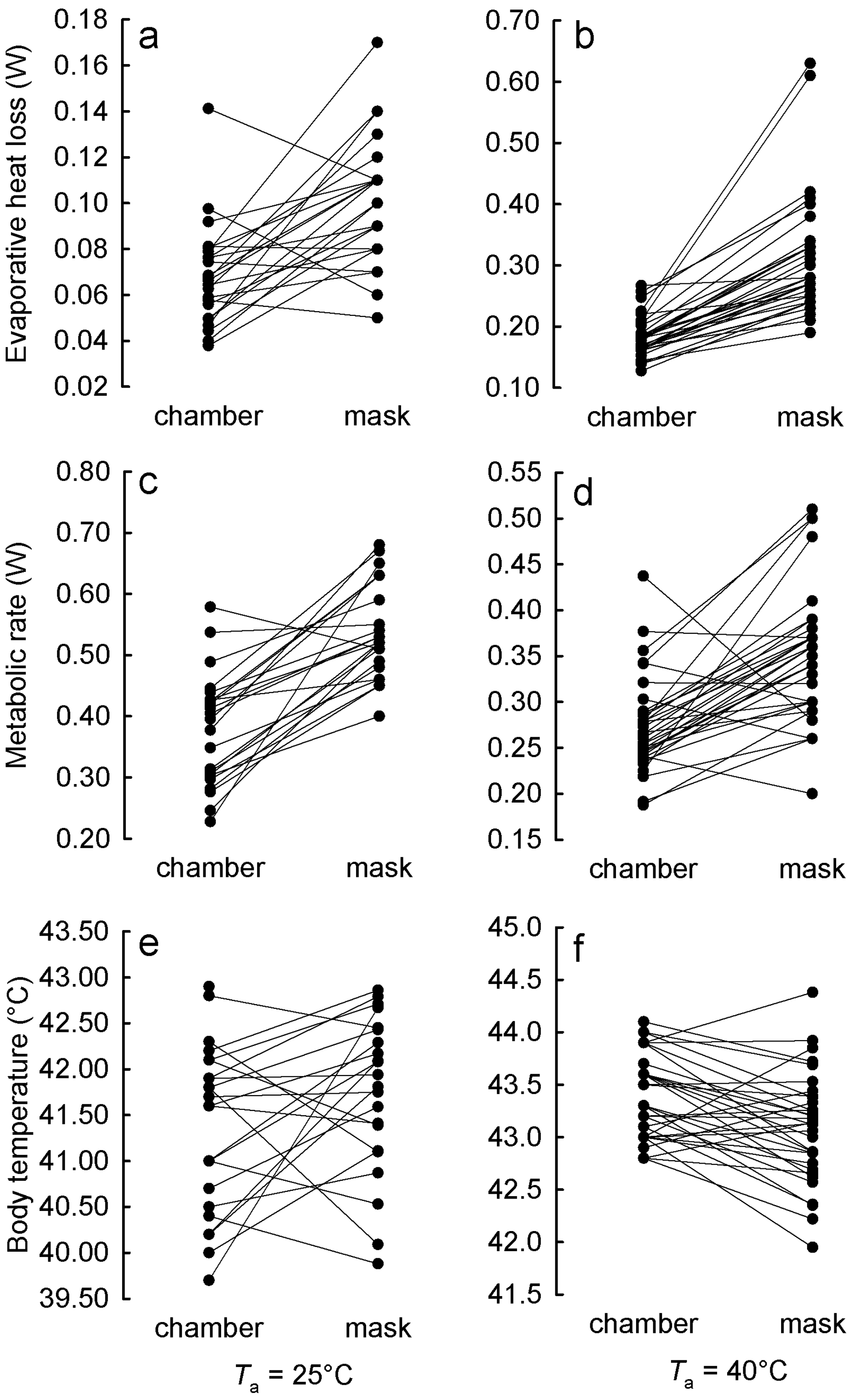

Fig. 2 Total evaporative heat loss (W; a, b), metabolic rate (W; c, d) and body temperature (°C; e, f) measured in zebra finches using whole-body (chamber) and mask respirometry at 25 and 40 °C. Total evaporative heat loss for mask measurements is a sum of respiratory and cutaneous evaporative heat loss. Lines connect measurements done on the same individuals
